# Building on Self-Determination Theory to Unravel the Motivational Drivers of Nurses and Rehabilitation Therapists in a Moroccan University Hospital: A Qualitative Study

**DOI:** 10.3390/nursrep16040116

**Published:** 2026-03-31

**Authors:** Abdellah Selmi, Zakaria Belrhiti

**Affiliations:** 1 Mohammed VI International School of Public Health, Mohammed VI University of Health and Sciences (UM6SS), Casablanca 82403, Morocco; 2 Public Health & Management Department, Mohammed VI Center for Research and Innovation (CM6RI), Rabat 10100, Morocco

**Keywords:** nursing, motivation, leadership, workload, organizational culture

## Abstract

**Background**: Nurses’ motivation is essential for ensuring the quality of care and workforce retention. Understanding the underlying psychological mechanisms is essential, particularly through Self-Determination Theory (SDT). This approach clarifies how work environments influence work engagement and performance. Existing research has explored SDT in various contexts. However, little is known about how contextual and organizational factors specifically impact nurses’ motivation in low- and middle-income countries such as Morocco. This study contributes to addressing this gap. **Objectives**: This study explores how organizational factors within a Moroccan university hospital influence the BPNs’ satisfaction in a Moroccan teaching university hospital. **Methods**: An exploratory qualitative case study was used. Purposive sampling was used to select 26 participants, including nurse managers (*n* = 7), rehabilitation therapists (*n* = 5), pharmacy technicians (*n* = 4), nurses at the Hematology Department (*n* = 4), nurses at the Emergency Department (*n* = 4), and nurses in the outpatient consultation unit of the Otorhinolaryngology Department (*n* = 2). Data collection was conducted from March to June 2023, following ethical approval. Data analysis followed Yin’s five-step process, incorporating deductive and inductive coding, within- and cross-case thematic analysis, and iterative explanation-building. **Results**: Identified motivation emerged as the most frequently reported type, although BPNs’ satisfaction varied. Rehabilitation therapists consistently reported high levels of autonomy, competence, and relatedness, which were attributed to transformational leadership, task specialization, and a supportive organizational culture. In contrast, nurses experienced role ambiguity, transactional leadership, excessive workloads, and limited autonomy in decision-making, all of which contributed to unmet BPNs. Performance evaluations and financial incentives were widely perceived as unjust. **Conclusions**: This study shows that in LMIC hospital settings, nurses’ motivation depends on organizational support for their BPNs, especially in resource-constrained environments. The significant disparities between professions within hospitals indicate that supportive environments with autonomy-supportive leadership, clear roles, fair evaluation, and adequate staffing are both achievable and essential for motivating and retaining nurses.

## 1. Introduction

Nurses play a pivotal role in health systems, representing 59% of health professionals and delivering 80% of care [1]. Low- and middle-income countries (LMICs) account for approximately 70% of the global nurse shortage, primarily due to low pay, challenging working conditions, inadequate protection, and low motivation [2]. Addressing these issues through targeted motivational interventions is a critical priority for strengthening health systems in LMICs [3]. Implementing effective motivational strategies enhances retention and overall health system performance [4,5].

Self-determination Theory (SDT) posits that people are growth-oriented and motivated by three Basic Psychological Needs (BPNs): autonomy, competence, and relatedness [6]. Meeting these needs supports wellbeing and autonomous motivation [7] (Supplementary File S1). The theoretical framework served as the primary basis for developing the interview guide and organizing the initial deductive analysis.

Motivation exists on a continuum, ranging from amotivation to intrinsic motivation, including forms of extrinsic motivation. Supportive work environments strengthen nurses’ BPNs. In contrast, toxic environments weaken them [7]. Transformational leadership encourages intrinsic motivation and transactional leadership can reduce autonomy and engagement [8].

Morocco faces a critical shortage of nurses, with only 0.9 nurses per 1000 inhabitants [9]. Nurses work under heavy workloads, high stress, and burnout, and have limited opportunities for advancement [10]. Transactional leadership and medical dominance reduce nurses’ autonomy and motivation [11].

In LMICs settings, nurses’ motivation is influenced by both positive and negative factors. Addressing demotivating elements, such as unclear job expectations [12], resource scarcity, characterized by shortages of medical supplies and equipment [12] and a role–competency mismatch occurs when nurses are required to perform tasks that exceed their qualifications [13], and health sector underinvestment constrains the graduation, employment, and retention of nurses within the health system [14]. This is why it is essential to enhancing motivating factors, including opportunities for professional development and recognition, contributes to the establishment of a high-quality health system [15]. Several factors contribute positively to nurses’ competency in ensuring patient safety, including their intrinsic motivation [16]. However, this motivation can be impacted by limited resources and the quality of supportive supervision [17]. Additionally, the type of hospital plays a role, as referral hospitals tend to handle higher patient volumes and increased workloads [18]. Unfairness in human resource management, particularly regarding financial conditions, also affects nurses’ motivation [19].

SDT offers a framework for understanding motivation, positing that social context does not influence behavior directly. Instead, its effects are mediated through the BPNs’ satisfaction or frustration and the subsequent quality of motivation [20,21]. However, limited qualitative research has explored how organizational factors affect nurses’ BPNs in LMIC hospital settings [22]. A qualitative approach is specifically designed to address the “what,” “how,” and “why” of human behavior [23]. Also, in Morocco, most studies have used quantitative methods to examine structural factors affecting nurses’ motivation. These factors include complex leadership styles, recognition, working conditions, dissatisfaction with salaries and remuneration, and poor communication [10]. Using SDT, this qualitative study examines how organizational factors within a Moroccan university hospital influence the BPNs’ satisfaction. This subsequently shapes whether nurses and rehabilitation therapists develop autonomous or controlled motivational profiles and regulation types.

## 2. Materials and Methods

### 2.1. Study Design and Setting

This study was conducted in a Moroccan public university hospital from March to June 2023. An exploratory qualitative study utilizing a multiple embedded case study design was conducted to provide a comprehensive understanding of the complex social phenomena influencing nurses’ motivation [24]. The primary case is the university hospital, within which specific departments, such as Emergency, Hematology, and ENT, and professional groups (e.g., kinesitherapeuts, general nurses) both serve as embedded units of analysis. These departments were chosen because they represent distinct medical specialties and treat varying patient pathologies, illustrating how diverse departments contribute to the overall institutional context.

### 2.2. Participants and Sampling

Purposive sampling was employed to recruit a diverse sample of healthcare professionals to ensure variation across clinical departments, specialties, and hierarchical positions [24]. Initial participants were asked to recommend colleagues, and the recommended individuals were subsequently contacted to participate. Inclusion criteria required participants to be licensed professionals with at least two years of service, working in direct patient care or management roles within the hospital. Exclusion criteria ruled out physicians and other healthcare professionals. The final sample consisted of 26 participants, including hematology nurses (*n* = 4), pharmacy technicians (*n* = 4), rehabilitation therapists (*n* = 5; physiotherapists and speech therapists), an otorhinolaryngology outpatient nurse (*n* = 2), emergency department nurses (*n* = 4), and nurse managers (*n* = 7). Four eligible nurses declined participation due to time constraints, and one was on leave.

### 2.3. Data Collection

Data were collected through individual, face-to-face, semi-structured interviews using a pre-tested guide developed from SDT constructs, focusing on the BPNs (Supplementary File S2). This guide ensured the investigation of the BPNs and key organizational factors across all participants and included a section on sociodemographic and professional characteristics. One of the interview questions was, “Can you tell me about your role in decision-making within your department, particularly regarding patient care?”. The interviews explored themes such as autonomy, competence, relatedness, leadership styles, and performance evaluation. Interviews, conducted in French by the first author, lasted approximately 40 min, were audio-recorded, and transcribed verbatim manually to maintain closeness to the data. Transcripts were then returned to participants electronically for verification through a process known as member checking. Selected excerpts were translated into English for publication by the authors with careful attention to preserving conceptual meaning and contextual nuance. Field notes were initially recorded as jottings and later expanded into full sentences after each interview. This process aimed to capture the workspace layout and reflect on emerging themes. Data saturation was achieved through the principle of redundancy, indicating that little new information was uncovered and that no additional themes emerged at two levels: within analysis units and across the entire case. All embedded units revealed stable, recurring patterns and relationships. A significant motivational contrast between nurses and rehabilitation therapists has become well-understood. An exception was observed for the Otorhinolaryngology outpatient nurses, as only two participants were in that group. For example, the findings consistently indicated conditions that supported the BPNs’ satisfaction with rehabilitation therapists, following Yin’s case study methodology [24].

### 2.4. Data Analysis

Data analysis adhered to Yin’s five-phase process for case studies, which facilitated iterative explanation-building and cross-case synthesis [24]. The process included:

1. *Compilation:* A structured database was created comprising all interview transcripts, with data uniformly formatted in tabular form, with one column corresponding to each participant. Field notes were also integrated alongside participant details.
2. *Disassembling:* The compiled data were organized into a tabular format based on SDT categories using deductive coding. An initial coding tree was developed (Supplementary File S3) and refined through iterative discussions among the authors. Coding was performed by the first author and validated by the second author to ensure consistency. By reviewing the coding structure, we assessed the alignment between the codes, data excerpts, and emerging interpretations. Any discrepancies were discussed with the first author until a consensus was reached. This tool served as the main framework for analysis, using a deductive coding approach based on the core BPNs. In parallel, inductive coding was conducted to identify emergent themes, such as role ambiguity and procedural injustice.
3. *Reassembling:* Performing within-unit analysis to develop thematic narratives for each professional group and identify recurring patterns. Then performing cross-unit analysis using thematic tables to compare similarities and differences across professional groups and departments.
4. *Interpreting:* Building on identified patterns and using SDT as our theoretical lens, we developed explanatory accounts. We examined how BPNs’ autonomy, competence, and relatedness were influenced by organizational factors, such as transformational versus transactional leadership and financial incentives. These factors served as either need-supportive or need-thwarting contexts. We also examined and identified patterns in forms of motivation for each professional group.
5. *Concluding:* In this final phase, we consolidated the findings from our embedded analysis units into a clear explanation that addresses the study’s objectives.

### 2.5. Trustworthiness and Rigor

Methodological rigor was maintained by adhering to the Consolidated Criteria for Reporting Qualitative Research guidelines [25]. Strategies included prolonged engagement in the field, triangulation of interview data with field notes, member checking of transcripts, and consensus discussions among authors on coding. Thick description of the context and purposive sampling further support the credibility and transferability of the findings.

### 2.6. Ethical Considerations

The study received ethical approval from the Ibn Rochd University Hospital Ethics Committee (Protocol No. 56/2023, dated 30 January 2023). The Participant Information Sheet (Supplementary File S4) and Informed Consent Form (Supplementary File S5) were designed to ensure fully informed, voluntary participation, thereby fostering an environment where participants felt secure in providing candid, in-depth accounts. The anonymized list of participants (Supplementary File S6) provides a detailed overview of the sample’s composition (e.g., specialty, seniority, education). This transparency supports the assessment of the study’s transferability and allows readers to contextualize the findings. Audio recordings were managed in compliance with Moroccan Law No. 09-08 on personal data protection and the principles of the Declaration of Helsinki [26]. All recordings were securely deleted upon completion of the analysis.

### 2.7. Data Availability Statement

The full anonymized interview transcripts and coding framework supporting this study are retained by the authors. These materials are not publicly available due to privacy and ethical restrictions but may be provided upon reasonable and justified request to the corresponding author.

## 3. Results

Three key themes emerged: substantial differences in BPNs’ satisfaction between nurses and rehabilitation therapists; the pivotal influence of organizational factors, particularly workload, role clarity, and leadership styles on BPNs’ satisfaction; and the persistence of identified motivation as the predominant form across all professional groups, regardless of these contextual disparities.

### 3.1. Characteristics and Demographics of Participants

The study included 26 participants, with 69.2% identifying as women. The mean age was 31.2 years (standard deviation 6.2), and the average professional experience was 9.5 years (standard deviation 5.9). Most participants held a bachelor’s degree (88.5%), 3.8% had a master’s degree, and 7.7% were licensed practical nurses (Table 1).

### 3.2. Motivation Forms

Motivation was predominantly characterized by autonomous motivation with identified regulation, which signifies that it occurs when an individual engages in an activity because they consciously value its importance or usefulness, even if the activity itself is not inherently enjoyable. A minority of participants were motivated by controlled motivation, specifically by external regulation, namely, financial constraints.

Similar to most nurses, those working in high-pressure settings, such as the hematology department, reported a strong motivation grounded in a desire to help patients. One nurse stated, “*The only thing that motivates me is helping people who need care. That is the only thing, nothing else, nothing else…*” (P22).

Also, the rehabilitation therapists evoked the same form of regulation. One of them stated: “*I know how important rehabilitation is in helping patients recover their autonomy and quality of life […] I truly believe it makes a meaningful difference, even if it’s not always easy.*” (P4).

In contrast, nurses working in departments such as the Ear, Nose, and Throat outpatient Department Nurse expressed externally regulated motivation: “*I come to work mainly for my children. I need to earn money to support them.*” (P17).

### 3.3. Basic Psychological Needs’ Satisfaction

Autonomy satisfaction was higher among rehabilitation therapists who made clinical decisions and organized their work. One affirmed this: “*The Ear, Nose and Throat specialist refers the patient to me, and that is it. I take care of the patient from A to Z… I’m the one who establishes the protocol…*” (P3). Elsewhere, nurses reported limited autonomy due to physician reliance, medical dominance, and hierarchical culture, and nurse managers likewise had little decision-making autonomy. One nurse manager stated, “*… we’re no longer invited to staff meetings; we’re just informed of the decisions… we simply endure whatever the higher-ups decide…*” (P10). Another participant added, “*As nurses, we must follow the doctors’ directives. We don’t have decision-making power; it’s the doctor who says what needs to be done, and we are responsible for doing it. Our job is to carefully carry out what they tell us. Our main role is to respond to medical orders, making sure we follow them rigorously…*” (P21).

Competence satisfaction varied by role and department: rehabilitation therapists showed strong self-efficacy, supported by opportunities and recognition; Hematology nurses reported low self-efficacy and distress from cancer-care outcomes. One nurse added: “*Unfortunately, we cannot guarantee good results, because healing is never certain. Many patients do not survive.*” (P22).

While pharmacy technicians showed strong technical self-efficacy, they also had lower self-efficacy regarding negative patient outcomes, which often caused additional psychological distress. One of them stated: “*I think I’m effective when preparing treatments, especially in oncology, where every dose must be precise and tailored to the patient. …We do everything we can, but recovery doesn’t always happen. Many patients, despite the treatments, end up passing away. …The rest is beyond my control.*” (P21).

Another pharmacy technician reported inequitable training opportunities. “*Access to scientific conferences is important for upgrading our skills and keeping up with medical advances, but it remains unequal. Some attend, others don’t…*” (P9).

Rehabilitation therapists cited continuing education and flexible work arrangements as enabling hyper-specialization and strengthening self-efficacy. “*My internship was made possible thanks to our department head’s initiative…in France. This experience enabled me to improve my skills. We have organized our work so that each team member handles a group of pathologies. This allows us to develop expertise…*” (P5).

In the emergency department nurses demonstrated strong self-efficacy and performed medical tasks beyond their scope of practice. One of them said: “*…Here in the emergency department, we handle patients without issue. We take care of what needs to be done and sometimes even more, like performing medical procedures when necessary…*” (P23).

Nurse managers showed self-efficacy and adaptive leadership in complex, volatile, high-pressure environments. One said: “*…We know how to handle issues, whether between colleagues or with patients. We’re here to ensure that patients coming to the hospital are treated with dignity…*” (P12).

Rehabilitation therapists, hematology nurse, Ear, Nose, and Throat outpatient department nurses and nurse managers described positive climates, strong cohesion, clan culture and team spirit. One reported: “*…We work like a small family, a close-knit team. If someone is unavailable, one of us takes care of their patients… we solve our problems among ourselves, without needing anyone’s intervention.*” (P4). Another stated: “*…I strive to manage equipment, staff, and medications efficiently to better care for our patients. Despite the constraints, I always try to maintain high levels of motivation and commitment within my team… my effectiveness lies in the team’s overall effectiveness*” (P15).

In contrast, high-pressure emergency department often saw nurses reporting limited solidarity. One participant stated: “*Unfortunately, there’s a lack of solidarity among emergency nurses. …for example, during tense periods in some workstations, nurses with lighter workloads should step in to help.*” (P24).

Conflictual dynamics often reflected excessive workload alongside procedural injustice. One nurse stated: “*We work here like machines, we provide care, but we don’t get any other benefits compared to other nurses working quietly in departments like Ear, Nose and Throat or ophthalmology*.” (P26).

A pharmacy technician saw poor cohesion due to conflict and intergenerational gaps: “*The older nurses used to do everything prep, administer, monitor, support, often with limited resources. Today, our role as pharmacy technician is more focused and technical. However, when we ask for better conditions, it’s sometimes misunderstood. We don’t always share the same work habits or expectations, which can lead to tension*.” (P21).

### 3.4. Leadership Styles

Leadership varied by department: nurses primarily experienced transactional leadership, whereas rehabilitation therapists reported transformational leadership characterized by inspiration, respect, listening and trust. As one therapist noted, “*As speech therapists, we have a great relationship with our head department. There is genuine mutual trust between us. He actively encourages us to give our best, to improve ourselves…that motivates us to improve our workplace.*” (P4).

Nurse managers reported non-participative governance, hierarchical and bureaucratic, with few opportunities for staff involvement or discussion of hospital management. One said: “*We have few opportunities to participate in decision-making or to discuss how the hospital is managed. Decisions are often imposed… we would like more collaboration to move things forward.*” (P10).

### 3.5. Financial Incentives and Performance Evaluation

All interviewees considered the financial incentive system inequitable. One participant stated: “*The bonus system is completely unbalanced. Some get bonuses that don’t match the effort and risks we take, while others who do twice the work get little to nothing. It’s just not fair*.” (P26).

Most participants perceived performance evaluations as lacking objectivity. One participant said: “*The annual bonus rating is based on personal impressions; it’s not the work accomplished that is taken into account, but the relationship with the head department*.” (P20).

Moreover, a nurse manager deemed evaluation, even if subjective, the only means of correcting some nurses’ inappropriate behaviors. One said: “*The only way left for us to correct behavior and ensure that nurses in the departments follow the rules is the rating we give them at the end of each year.*” (P15).

### 3.6. Role Ambiguity

Most participants, except rehabilitation therapists, reported role ambiguity, lowering autonomous motivation and heightening perceived workload. This ambiguity resulted from the informal expansion of tasks, which served as an adaptive response to persistent understaffing and elevated patient volumes. In the Moroccan context, this phenomenon represents a regulatory gray zone in which professional boundaries are neither clearly defined nor consistently enforced. An emergency department nurse stated: “*The tasks are not clearly defined, which leads me to perform duties not part of my nursing role, including medical procedures. I often find myself overwhelmed with administrative and logistical tasks… should be assigned to specific profiles.*” (P25).

Additionally, role ambiguity was identified between licensed practical nurses and nurses. This ambiguity arises from a weakly defined scope of work, resulting in licensed practical nurses frequently performing nursing care similar to those of their nurse colleagues. Rehabilitation therapists articulated a clear and precise understanding of their professional role, as demonstrated by one participant: “*For now, I do nothing other than my work as a physiotherapist, nothing more*.” (P4).

### 3.7. Workload and Staffing

Acute staffing shortages and a perceived high workload often exacerbated nurses’ dissatisfaction. One of them said: “*We are always short-staffed, which means we have too many patients to care for. The situation gets even worse during summer and school holidays*.” (P20). A pharmacy technician added: “*There aren’t enough of us to handle the workload. Sometimes, we’re left alone to manage multiple complex chemotherapy. It’s exhausting, and we feel like no one really understands what we go through in the department*” (P9).

In contrast, rehabilitation therapists with well-defined, task-specialized roles in positive environments perceived staffing as adequate and workload manageable. One noted: “*Honestly, the workload is manageable, and we have enough staff for the number of patients we handle.*” (P4).

### 3.8. Organizational Subcultures

In hematology department, excessive hierarchy, medical dominance and strict rules, alongside distress from adverse cancer outcomes, lowered nurses’ and pharmacy technicians’ motivation and well-being. One nurse said, “*We are here to support patients through one of the most difficult moments of their lives. It’s an extremely heavy emotional burden to carry. …, I am confronted with suffering, fear, and death in the eyes of the patients.*” (P20).

Emergency department nurses often felt strained by unpredictable, complex, volatile patient flows, disorganization and staff shortages. One nurse said: “*The pace of work is intense. Sometimes we lack the resources to handle everything, and managing all becomes difficult. The team is determined to provide quality care, even in these conditions.*” (P26).

In the Ear, Nose, and Throat outpatient department, nurses reported extreme stress, verbal and non-verbal violence tied to disorganization and mass patient inflow, sometimes escalating to verbal aggression. One of them said: “*We are constantly being insulted, sometimes to the point where the police must intervene. We know it’s mostly symbolic because, most of the time, things are settled amicably…*” (P17).

In contrast, rehabilitation therapists described a calm, peaceful, privileged environment. One therapist noted: “*It’s an advantage to work here. It allows me to fully dedicate myself to my patients, whom I’ve known for years… Honestly, I can’t imagine being part of a toxic team…*” (P1).

**Figure 1:**
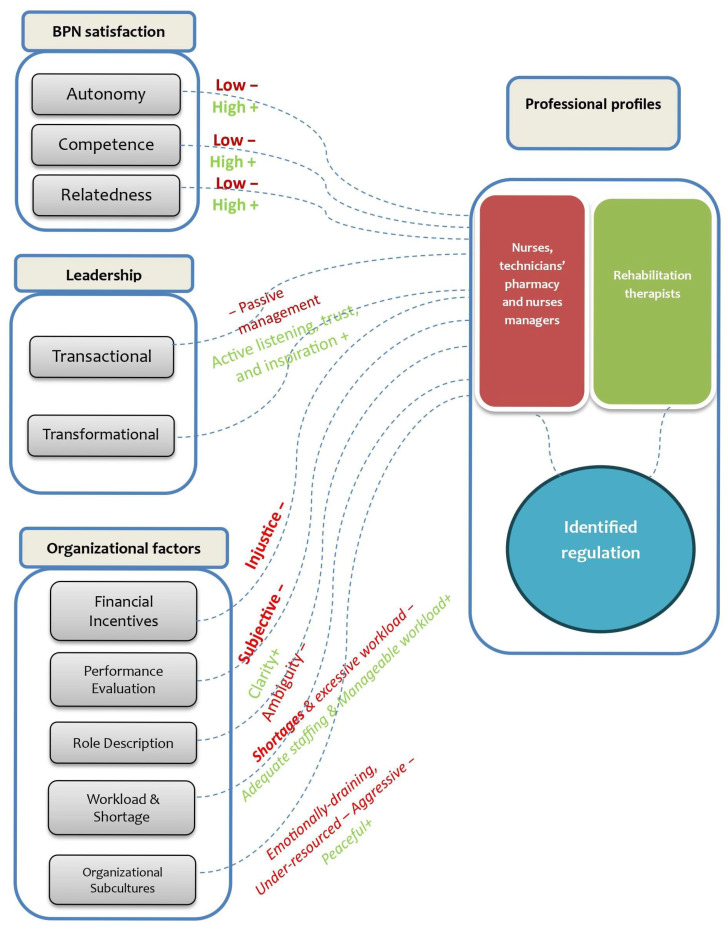
The complexity of nurses’ motivation.

BPNs’ satisfaction shaped motivation. Rehabilitation therapists showed identified regulation under high autonomy, competence and transformational leadership. Despite ambiguity, heavy workloads and transactional leadership, nurses showed identified regulation (Figure 1).

Figure 1 demonstrates how organizational factors influence the BPNs’ satisfaction, with need-thwarting conditions for nurses depicted in red and need-supportive conditions for rehabilitation therapists in green. The model indicates that these BPNs function as central mediators of motivational quality, which spans from controlled to autonomous regulation. Identified regulation emerged as the most prevalent motivational form across both groups. However, its manifestation varies considerably between contexts: in need-thwarting environments, it appears fragile and pressured, whereas in need-supportive environments, it is experienced as more volitional and integrated. The complexity of nurses’ motivation refers to the dynamic interplay between the fulfillment of basic psychological needs (autonomy, competence, and relatedness) and individual characteristics such as values, expectations, and professional identity. This motivation is continuously shaped by interactions with leadership practices and organizational contexts, making it a fluid and context-dependent process rather than a fixed individual attribute.

## 4. Discussion

This study examines the complex motivational landscape within a Moroccan hospital, using SDT as an analytical framework. The results indicate that organizational contexts variably support or hinder the BPNs of nurses and rehabilitation therapists, resulting in distinct motivational profiles despite shared professional values.

### 4.1. SDT-Driven Analysis of Motivational Heterogeneity

The main finding that identified regulation predominates across groups despite differences in BPNs’ satisfaction is indicative of a complex internalization process [27]. It signifies a partial and fragile internalization of the prosocial value of helping patients. This form of motivation persists as personally important despite the need-thwarting environment, but lacks the full integration and volition of more autonomous forms [27]. The dominance of identified regulation can be linked to various organizational factors that hinder the BPNs’ satisfaction among nurses. These factors include: transactional leadership, an unfair financial incentive system, performance evaluations that lack objectivity, and unclear job roles. Additionally, acute staffing shortages, a perceived heavy workload, and organizational cultures characterized by excessive hierarchy contribute to significant stress for nurses, further diminishing their BPNs’ satisfaction. Nurses engage in their work because they personally value its importance and see it as aligned with their goals, rather than due to external rewards or pressure [28].

In contrast, rehabilitation therapists demonstrate what SDT characterizes as identified regulation [29]. Their high BPNs’ satisfaction creates conditions in which work activities align seamlessly with both professional identity and personal values [30]. Their professional identity, rooted in recognized expertise, and task specialization, directs motivation toward intrinsic goals, including quality of care and perceived social usefulness. Thus, professional identity serves as an internalization factor, enabling the acceptance of work demands as personally meaningful and sustaining identified regulation among rehabilitation therapists [29].

### 4.2. The BPNs as Differential Mediators

#### 4.2.1. Autonomy: The Critical Divider

SDT posits autonomy as the experience of volition [27]. Our findings sharply differentiate the two groups along this dimension. Rehabilitation therapists’ autonomy was supported through clinical autonomy, protocol establishment, and work organization, all autonomy-supportive features that align with SDT’s emphasis on choice and initiative [7]. Conversely, nurses experienced autonomy frustration through multiple channels: medical dominance, bureaucratic constraints, and transactional leadership [11,31]. This systematic thwarting of autonomy needs represents what SDT identifies as a primary pathway to controlled motivation and diminished well-being [8].

#### 4.2.2. Competence: Specialization Versus Generalization

Competence, the need to feel effective and master challenges [27], manifested differently between groups. Rehabilitation therapists enhance their competences by cultivating task-specific skills through specialization and ongoing education [32]. Nurses, however, faced competence-undermining conditions: tasks misaligned with training (especially for those with higher education), inadequate staffing that prevented mastery experiences, and cancer care outcomes that inherently undermined feelings of effectiveness [33]. This competence frustration explains the distress and perceived low self-efficacy reported despite their technical skills.

#### 4.2.3. Relatedness: Clan Culture Versus Fragmented Solidarity

Relatedness, the need for connection and belonging [27], varied significantly between the groups. Relatedness helps rehabilitation therapists feel more connected to their professional roles and fosters a sense of belonging and family-like cohesion [34]. Unmet relatedness needs among emergency department nurses may contribute to a decline in the overall quality of the work environment, thereby increasing burnout and job dissatisfaction [35]. Nurse managers’ isolation from decision-making hinders their sense of belonging [36].

### 4.3. Organizational Factors as Need-Supportive or Need-Thwarting Contexts

SDT emphasizes that motivation arises from person-context interactions [27]. Our analysis shows how specific organizational factors serve as either need-supportive or need-thwarting elements.

#### 4.3.1. Leadership Styles

Transformational leadership among rehabilitation therapists offered autonomy support, competence feedback and relatedness through trust, aligning with SDT’s concept of need-supportive leadership [7]. This leadership style fosters autonomy, increases job satisfaction, and cultivates a supportive work environment, collectively contributing to improved patient outcomes [37]. Transactional leadership for nurses represented a controlling context that SDT associates with reduced autonomy [8].

#### 4.3.2. Financial Incentives and Performance Evaluation

Perceived injustice in performance evaluations and financial incentives stems from inadequate compensation and inter-organizational disparities, while a ‘complacency’ effect occurs when high-performing employees become demotivated because less-productive colleagues receive similar rewards due to a lack of objective evaluation [38]. The current performance evaluation represents controlling feedback that undermines autonomy and competence [39].

#### 4.3.3. Work Design

Rehabilitation therapists’ specialized roles created job crafting opportunities that enhanced autonomy and competence [40]. Nurses’ ambiguous roles and excessive workloads created job strain that systematically thwarted all three BPNs [35]. In the Moroccan context, Nursing Practice Law No. 43-13 does not establish a legally defined scope of nursing practice, as it fails to clearly delineate authorized nursing acts [41]. Unlike physicians, nurses do not have an independent regulatory body, which limits their capacity for professional self-governance. A similar situation exists between licensed practical nurses and general nurses, who deliver identical nursing care without clear role differentiation, a condition originating from a time when general nurses were in short supply.

#### 4.3.4. Workload and Staffing

Chronic understaffing and excessive workloads constitute an environmental pressure that directly thwarts BPNs [42]. Understaffing and high workloads undermine nurses’ clinical autonomy and create a disconnect between health policy and actual practice [43]. Extended shifts contribute to burnout, which can compromise patient safety and care quality [44], Understaffing increases job dissatisfaction by creating unfavorable work environments due to a lack of necessary support, which hinders the provision of quality care [45]. In contrast, rehabilitation therapists’ manageable workloads and adequate staffing created a need-supportive environment, enabling the satisfaction of their BPNs [46].

### 4.4. Theoretical Contribution to SDT in Healthcare Contexts

#### 4.4.1. Contextualizing BPN Satisfaction in LMICs Hospitals

The work context is a factor. When the context is supportive and nurtures nurses’ BPNs, these needs are satisfied, fostering autonomous motivation, which leads to adaptive outcomes such as higher job satisfaction, stronger work engagement, and improved job performance. In contrast, when the context is need-thwarting and frustrates these BPNs, such frustration tends to generate controlled forms of motivation, which are associated with maladaptive outcomes, including elevated stress, burnout, and turnover intentions [47].

#### 4.4.2. Professional Identity and Gender Dynamics

It affects the job satisfaction of rehabilitation therapists. It is shaped by personal experiences and values, professional education including academic training and clinical placements and the broader profession [48]. Nurses’ medically subordinate roles decrease the BPNs’ satisfaction [49].

Although the limited number of male participants in our study (*n* = 8) limits our ability to make robust gender-based comparisons, the identified regulation predominance among our majority of female sample (69.2%) reflects the global trend of increasing female representation in nursing and the gendered socialization towards caregiving [1]. Existing literature indicates that women in the nursing profession often prioritize prosocial values and social recognition, which aligns with identified regulation [50]. In contrast, male nurses may show greater sensitivity to financial incentives such as status and direct compensation [51]. Furthermore, transformational leadership has been shown to enhance motivation across genders [52]. Additionally, perceptions of organizational systems can differ, with female nurses often holding more positive views of the efficacy of performance evaluation compared to their male colleagues [53].

#### 4.4.3. Subcultural Variations Within Single Organization

It serves as a significant moderator of BPNs’ satisfaction and motivational regulation among professional groups in hospital environments [54].

At the national level, Moroccan culture is characterized by collectivism, where social cohesion, and family loyalty take precedence over individual interests [55]. This high power distance reflects acceptance of hierarchical structures in both social and professional settings, fostering loyalty and respect for seniority [50]. From the perspective of SDT, such a culture may enhance the need for relatedness but weaken autonomy and the motivational impact of financial incentives. Leadership is generally perceived as transactional. In contrast, individualist cultures value autonomy and personal achievement, which can enhance the effects of autonomy-supportive leadership and professional development [56,57].

These results are consistent with Bronfenbrenner’s ecological systems model to argue that just as distal cultural norms trickle down to shape proximal need satisfaction, so too do institutional hierarchies and managerial practices constitute a motivational ecology whose consequences impact individual BPN levels [54].

### 4.5. Limitations

This study has several limitations. First, the primary author’s professional identity as a nurse may have influenced interpretation. To address this potential bias, regular, structured debriefings and reflexive discussions were conducted between the two authors, during which interpretations and analytic decisions were critically examined for subjectivity [58]. These meetings facilitated reflexivity by explicitly considering how the author’s professional background might shape data analysis and theme development. Clear boundaries were stated to address power dynamics and avoid managerial discourse. The researcher acted solely as a PhD student, and reflective practice was consistently observed throughout data collection and analysis. We maintained subjective neutrality when interviewing, collecting data, and analyzing data. No role has been played by hospital management in the design nor in participant selection, as these were guided by theoretical assumptions. Although positioned as an insider researcher, reflexive practices and continuous critical self-awareness were used to mitigate potential biases and maintain analytical neutrality. Particular attention was paid to existing power dynamics, ensuring that data collection and interpretation remained balanced, respectful, and independent of institutional hierarchy.

An audit trail was maintained to document analytic choices, and coding was cross-checked with the second author, who provided independent perspectives to support analytic rigor [59]. Social desirability, confirmation, and history biases may also have influenced participants’ responses, potentially suppressing extrinsic financial motives while highlighting altruistic narratives [60]. However, trust and triangulation between different data sources were maintained.

Another limitation is the sample size, which consists of only 26 participants. Additionally, the single-case design, focusing on just one university hospital, restricts the statistical generalizability of the findings. Furthermore, including only two nurses from the Ear, Nose, and Throat outpatient department limits the diversity of perspectives within this subgroup, as the unit comprises only these two nurses.

## 5. Conclusions

This study offers a new and context-rich perspective on healthcare worker motivation in LMICs by applying SDT within a Moroccan public hospital. The findings indicate that nurses’ motivation is closely tied to their BPNs for autonomy, competence, and relatedness. Factors such as transactional leadership, chronic understaffing, excessive workloads, subjective performance evaluations, and perceived unfairness in financial incentives significantly impact this motivation. Based on our findings, we recommend that hospital managers and policymakers implement the following interventions; provide autonomy-supportive leadership training to involve nurses in decision-making, establish clear and equitable role definitions to minimize ambiguity and prevent unfair task expansion, create transparent, competency-based evaluation systems to replace subjective performance evaluations, and facilitate structured team-building activities, and include staff in governance to strengthen feelings of relatedness. These targeted, evidence-based interventions address the core BPNs identified as crucial for maintaining motivation for healthcare workers in challenging hospital environments in LMICs.

Future research should evaluate these interventions using longitudinal designs across diverse hospital settings to strengthen evidence regarding their impact on nurse motivation and other healthcare professionals. Studies are required to assess the causal effects of specific need-supportive changes. Comparative case studies across various hospital types, such as public versus private and regional versus local institutions in Morocco and other LMICs are recommended. Additionally, future research should investigate how gender influences the satisfaction and motivational outcomes of BPNs. The dynamics of gender within healthcare hierarchies may affect nurses’ experiences in ways that have not been thoroughly examined in the existing literature.

## Figures and Tables

**Table 1:** Characteristics of participants.

Specialty	Participant, *n* (%)	Male, *n* (%)	Female, *n* (%)	Mean Age ± SD (Years)	Mean Seniority ± SD (Years)	Licensed Practical Nurse	Bachelor’s Degree	Master’s Degree
Rehabilitation therapists	5 (19.2%)	1 (20.0%)	4 (80.0%)	27.0 ± 1.0	4.0 ± 1.0	0 (0%)	5 (100%)	0 (0%)
Pharmacy technicians	4 (15.4%)	0 (0%)	4 (100%)	27.0 ± 0.8	6.0 ± 0.8	0 (0%)	4 (100%)	0 (0%)
Nurse managers	7 (26.9%)	4 (57.1%)	3 (42.9%)	42.0 ± 1.3	18.0 ± 1.3	0 (0%)	6 (85.7%)	1 (14.3%)
Nurses in outpatient consultation unit of the Otorhinolaryngology Department	2 (7.7%)	0 (0%)	2 (100%)	27.0 ± 1.4	4.0 ± 1.4	1 (50.0%)	1 (50.0%)	0 (0%)
Nurses at Hematology Department	4 (15.4%)	0 (0%)	4 (100%)	33.0 ± 0.8	10.0 ± 0.8	1 (25.0%)	3 (75.0%)	0 (0%)
Nurses at the Emergency Department	4 (15.4%)	3 (75.0%)	1 (25.0%)	28.0 ± 0.8	5.0 ± 0.8	0 (0%)	4 (100%)	0 (0%)
Total	26 (100%)	8 (30.8%)	18 (69.2%)	31.2 ± 6.2	9.5 ± 5.9	2 (7.7%)	23 (88.5%)	1 (3.8%)

## Data Availability

The data from the interviews are not available due to data protection and privacy considerations.

## Supplementary Materials

The following supporting information can be downloaded at https://www.mdpi.com/article/10.3390/nursrep16040116/s1. Supplementary File S1: Self-Determination Theory Concepts; Supplementary File S2: Interview Guide; Supplementary File S3: Initial coding tree; Supplementary File S4: Participant Information Sheet; Supplementary File S5: Informed consent form; Supplementary File S6: List of participants with code.

## Public Involvement Statement

No public involvement in any aspect of this research.

## Guidelines and Standards Statement

This study was conducted following the Consolidated Criteria for Reporting Qualitative Research (COREQ).

## Abbreviations

The following abbreviations are used in this manuscript:

SDT	Self-Determination Theory
BPNs	Basic Psychological Needs
LMICs	Low- and Middle-Income Countries
