# Supplementary material for: Building on Self-Determination Theory to Unravel the Motivational Drivers of Nurses and Rehabilitation Therapists in a Moroccan University Hospital: A Qualitative Study
Source: Nurs Rep. 2026 Mar 31;16(4):116. doi: 10.3390/nursrep16040116 (PMC13118901; doi:10.3390/nursrep16040116)
Supplement: Supplementary file 1 [file nursrep-16-00116-s001.zip › nursrep-4097939-supplementary.pdf]

**Supplementary file S1: Self-Determination Theory Concepts**

Self-Determination Theory constitutes a comprehensive macro-theory of human motivation, encompassing six interrelated mini-theories: Basic Psychological Needs Theory, Organismic Integration Theory, Cognitive Evaluation Theory, Causality Orientations Theory, Goal Contents Theory, and Relationships Motivation Theory [1].

**Autonomy:** This refers to the experience of volition and willingness, a sense of choice-making and being the initiator of one's own actions [1].

**Competence:** This refers to the experience of a sense of effectiveness, mastery, and accomplishment [2].

**Relatedness:** refers to the need to feel a sense of connection and belonging in relation to other members of one’s social environment [2].

Amotivation: This represents the complete absence of motivation or intention [2].

**Intrinsic Motivation:** this is the most autonomous type of motivation. It occurs when individuals engage in an activity for the interest and enjoyment they derive directly from the activity itself [2].

**Extrinsic Motivation:** This applies to behaviours that are primarily instrumental, being governed by the prospect of reward and punishment [3].

**Autonomous Motivation:** This typically comprises intrinsic motivation and identified regulation, and integrated regulation. It refers to acting volitionally and with a sense of choice and willingness [4].

**Identified Regulation:** This type of motivation is a relatively autonomous form of extrinsic motivation, where individuals consciously value a behavior and consider it personally significant, even if the activity itself is not inherently enjoyable [2].

**Integrated Regulation:** This is the most autonomous form of extrinsic motivation. It occurs when identified regulations become fully integrated with an individual's sense of self, values, and identity, making the behavior feel self-endorsed and aligned with who they are [2].

**Controlled Motivation:** it’s comprising introjected regulation and external regulation. It refers to acting with a sense of pressure and obligation, rather than a sense of volition, pleasure, or self-endorsement [2].

**Introjected Regulation:** This represents a controlled form of extrinsic motivation, where behavior is driven by internal pressures such as guilt, shame, or anxiety, rather than by genuine choice or personal valuation [2].

**External Regulation:** This is the most controlled form of extrinsic motivation, characterized by behaviors that are influenced by external demands, rewards, or punishments imposed by others or the environment [2].

**References:**


1. Ryan, R.M.; Deci, E.L. *Self-Determination Theory: Basic Psychological Needs in Motivation, Development, and Wellness*; Guilford Press: New York, 2017; ISBN 978-1-4625-2876-9.

2. Deci, E.L.; Ryan, R.M. Self-Determination Theory: A Macrotheory of Human Motivation, Development, and Health. *Canadian Psychology / Psychologie canadienne* **2008**, *49*, 182–185, doi:10.1037/a0012801.
3. Gagné, M.; Deci, E.L. Self-determination Theory and Work Motivation. *J Organ Behavior* **2005**, *26*, 331–362, doi:10.1002/job.322.
4. Deci, E.L.; Ryan, R.M. The "What" and "Why" of Goal Pursuits: Human Needs and the Self-Determination of Behavior. *Psychological Inquiry* **2000**, *11*, 227–268, doi:10.1207/S15327965PLI1104_01.

**Supplementary file S2: Interview Guide**

**Researchers Introduction**

Gender, Credentials, Occupations / Professional roles, Experiences

**Purpose of the interview:**

This research aims to explore the motivational drivers of nurses and rehabilitation therapists using SDT, focusing on organizational factors that influence motivation through BPNs

**Ethical considerations:**

The study received ethical approval and was conducted in accordance with the Declaration of Helsinki, ensuring respect for participant anonymity and confidentiality. All recorded and collected information is managed to prevent the disclosure of participant identities.

## Recording

I would like to record this interview to ensure better understanding and analysis of the information shared.

## General Information

- Email and phone:
- Age:
- Seniority:
- Educational level:
- Nursing specialty:

## Autonomy

- Q: Can you tell me about your role in decision-making within your department, especially when it comes to patient care?
- Q: How do you perceive this role?

## Competence

- Q: How do you perceive your effectiveness at work?
- Q: How do you perceive the collective effectiveness of your team?
- Q: Tell me about the continuing education or training you've taken part in?

## Relatedness

- Q: How would you describe the relationships between your colleagues?
- Q: What do you think about workplace violence or harassment?
- Q: How do you perceive the justice among colleagues in the workplace?

## Motivation regulation

Can you tell me what motivates you the most to come to work?

## Leadership

- Q: How would you describe the leadership style of your department head? Can you share any examples of how they manage or guide the team on a daily basis?

- Q: Can you tell me how well they listen to your concerns or suggestions, and how much you trust them in their role?

## Organizational factors

- Q: How do you evaluate your performance evaluation?
- Q: What are your thoughts on the annual bonus you receive?
- Q: What do you think about your job description?
- Q: How do you perceive the staffing level of nursing in your department?
- Q: How do you perceive your workload?

## Summary and Debriefing

During this interview, you provided very interesting and useful information.

Is there anything you consider important in relation to what we discussed that hasn’t been mentioned yet? If so, we still have time and can discuss it now.

Do you have any questions you would like to ask me?

# Supplementary file S3 : Initial coding tree

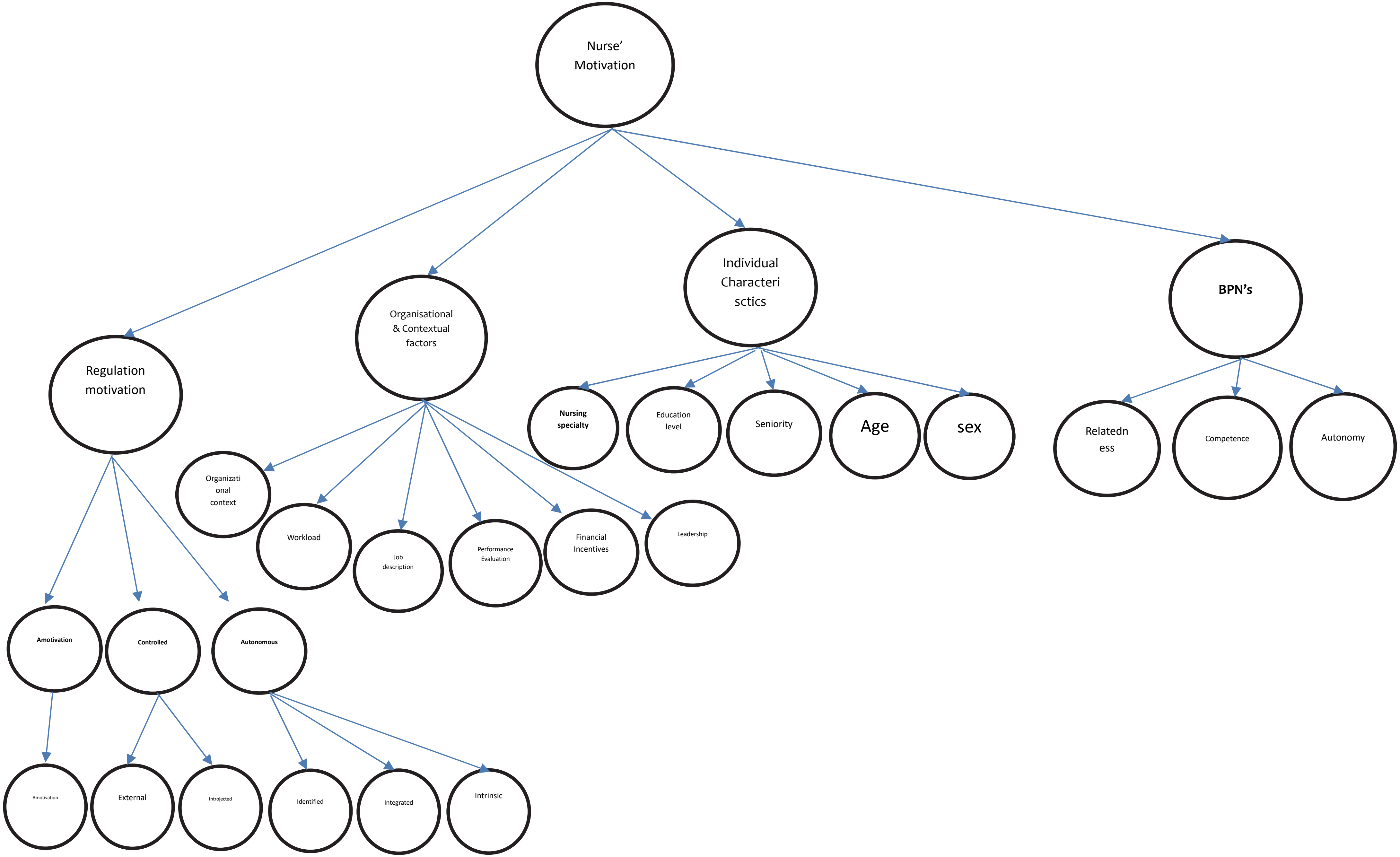

**Supplementary file S4 : Participant Information Sheet**

Dear Sir/Madam,

You are invited to review this information sheet regarding our research project on nurse motivation.

Researchers Introduction

Gender, Credentials, Occupations / Professional roles, Experiences

## Background to the Study

The nursing profession is central to healthcare systems, responsible for approximately 80% of direct patient care. In Morocco, a significant shortage of nursing personnel exists amidst high professional pressure, widespread burnout, and low job satisfaction.

## Research Objectives

This research aims to explore the motivational drivers of nurses and rehabilitation therapists using SDT, focusing on organizational factors that influence motivation through BPNs

## Methodology

Our research employs an exploratory qualitative approach based on a case study design. This methodology facilitates an in-depth understanding of the intricate relationships between nurse motivation, leadership styles, and organizational factors.

Data collection will be conducted through semi-structured interviews, each lasting an average of 40 minutes. These interviews will be audio-recorded and subsequently transcribed verbatim for analysis.

## Modality of Participation

Should you choose to participate, you will engage in an individual interview of approximately 40 minutes with a researcher, during which your professional experiences and perceptions of work motivation will be discussed.

Your participation is entirely voluntary, and you reserve the right to withdraw at any time without providing justification or incurring any negative consequences. Your confidentiality will be maintained, and your identity protected through the use of anonymous identification codes in all research documents.

## Importance of This Research

This study is anticipated to generate valuable knowledge that will inform strategies for human resource management in healthcare.

By participating, you will contribute to a deep understanding of the factors influencing nurse motivation, potentially leading to tangible improvements in working conditions and professional satisfaction.

## Anticipated Benefits for the Nursing Profession

This research aims to:

- Develop leadership and management strategies better tailored to the needs of nurses.
- Improve systems of incentives and professional recognition.
- Enhance the autonomy and acknowledgment of the nursing role within the healthcare system.
- Contribute to the reduction of staff turnover and the improvement of nurse retention.
- Promote a work environment more conducive to the satisfaction of fundamental psychological needs.

Ultimately, these improvements are expected to not only benefit nurses but also positively impact the quality of care and patient outcomes.

## For Further Information

Should you require further information regarding this study or have any questions, please do not hesitate to contact:

# Supplementary file S5 : Informed consent form

## Introduction

You are invited to participate in a qualitative research study on the motivation of nurses in the Moroccan hospital context. This document provides you with the necessary information to help you decide whether or not to take part. Please read it carefully, and feel free to ask any questions you may have before making your decision.

## Purpose of the Study

This research aims to explore the motivational drivers of nurses and rehabilitation therapists using SDT, focusing on organizational factors that influence motivation through BPNs

## Methodology and Procedure

If you agree to participate, you will be invited to take part in a semi-structured interview lasting approximately 40 minutes. The interview will be audio-recorded to ensure accurate analysis of your responses and will be conducted by a qualified researcher.

## Voluntary Participation and Right to Withdraw

Your participation in this study is entirely voluntary. You are free to accept or decline without having to justify your decision, and this will not affect you in any way. You may also withdraw from the study at any time, even after giving your consent, without facing any negative consequences.

## Confidentiality and Anonymity

All information collected in this study will be treated confidentially. Your identity will be protected through the use of identification codes (P1, P2, etc.) in all research documents and publications. Audio recordings will be securely stored and destroyed after data transcription and analysis.

## Potential Risks

This study involves minimal risk. You may experience slight discomfort when discussing difficult professional situations or complex workplace relationships. If this occurs, you are free to skip any question or stop the interview at any time.

## Potential Benefits

While there is no direct benefit for you, your participation will contribute to a better understanding of the factors influencing nurse motivation in Moroccan hospitals. The findings may help develop strategies to improve working conditions and job satisfaction for nurses, which could benefit you indirectly in the future.

## Dissemination of Results

The results of this study may be published in scientific journals or presented at conferences. In all cases, no identifying information will be disclosed.

## Financial Compensation

There is no financial compensation for participating in this study.

## Questions and Contact

If you have any questions about this study—before, during, or after your participation please feel free to contact the principal investigator:

## Consent

I, the undersigned, ____________________, hereby declare that:

- I have read and understood the information presented in this document.
- I have had the opportunity to ask all my questions and received satisfactory answers.
- I understand that my participation is voluntary and that I may withdraw at any time without justification.
- I agree to the audio recording of the interview.
- I freely consent to participate in this study under the conditions described above.

Participant’s signature: ____________ Date: ____________

Researcher’s signature: ____________ Date: ____________

This form is issued in two copies: one for the participant, and the other is kept by the researcher.

## Supplementary file S6 : List of participants with code

| Code | Specialty | Age (years) | Gender | Seniority Years | Level of education |
|---|---|---|---|---|---|
| P1 | Rehabilitation Therapist | 26 | F | 3 | Bachelor's Degree |
| P2 | Rehabilitation Therapist | 27 | F | 4 | Bachelor's Degree |
| P3 | Rehabilitation Therapist | 28 | F | 5 | Bachelor's Degree |
| P4 | Rehabilitation Therapist | 26 | F | 3 | Bachelor's Degree |
| P5 | Rehabilitation Therapist | 28 | M | 5 | Bachelor's Degree |
| P6 | Pharmacy technician | 26 | F | 5 | Bachelor's Degree |
| P7 | Pharmacy technician | 27 | F | 6 | Bachelor's Degree |
| P8 | Pharmacy technician | 28 | F | 7 | Bachelor's Degree |
| P9 | Pharmacy technician | 27 | F | 6 | Bachelor's Degree |
| P10 | Nurse Manager | 40 | F | 16 | Bachelor's Degree |
| P11 | Nurse Manager | 42 | F | 18 | Master's Degree |
| P12 | Nurse Manager | 44 | F | 20 | Bachelor's Degree |
| P13 | Nurse Manager | 41 | M | 17 | Bachelor's Degree |
| P14 | Nurse Manager | 43 | M | 19 | Bachelor's Degree |
| P15 | Nurse Manager | 42 | M | 18 | Bachelor's Degree |
| P16 | Nurse Manager | 42 | M | 18 | Bachelor's Degree |
| P17 | General nurses in outpatient consultation unit of the Otorhinolaryngology Department | 28 | F | 5 | Bachelor's Degree |
| P18 | General nurses in outpatient consultation unit of the Otorhinolaryngology Department | 26 | F | 3 | Licensed Practical Nurse |
| P19 | General nurse at the Hematology Department | 32 | F | 9 | Bachelor's Degree |
| P20 | General nurse at the Hematology Department | 33 | F | 10 | Licensed Practical Nurse |
| P21 | General nurse at the Hematology Department | 34 | F | 11 | Bachelor's Degree |
| P22 | General nurse at the Hematology Department | 33 | F | 10 | Bachelor's Degree |
| P23 | General nurse at the emergency Department | 27 | F | 4 | Bachelor's Degree |
| P24 | General nurse at the emergency Department | 28 | M | 5 | Bachelor's Degree |
| P25 | General nurse at the emergency Department | 29 | M | 6 | Bachelor's Degree |
| P26 | General nurse at the emergency Department | 28 | M | 5 | Bachelor's Degree |

**Total participants:** 26
**Total women:** 18
**Total men:** 8
**Average age:** 31 years

**Level of education:**
Licensed Practical Nurse: 2
Bachelor's Degree: 23
Master's Degree: 1
